# *N*-(4-Meth­oxy-2-nitro­phen­yl)-*N*-(methyl­sulfon­yl)methane­sulfonamide

**DOI:** 10.1107/S160053681202483X

**Published:** 2012-06-13

**Authors:** Sammer Yousuf, Hina Siddiqui, Rabia Farooq, M. Iqbal Choudhary

**Affiliations:** a H.E.J. Research Institute of Chemistry, International Center for Chemical and Biological Sciences, University of Karachi, Karachi 75270, Pakistan; b Department of Biochemistry, Faculty of Sciences, King Abdul Aziz University, Jeddah 21589, Saudi Arabia

## Abstract

In the title compound, C_9_H_12_N_2_O_7_S_2_, the nitro substituent is slightly twisted from the benzene ring [dihedral angle = 14.69 (10)°]. The mol­ecular geometry is stabilized by intra­molecular C—H⋯O hydrogen bonds, forming *S*(6) ring motifs. In the crystal, molecules are linked by C—H⋯O hydrogen bonds into layers parallel to (10-2).

## Related literature

For the biological activities of sulfonamides, see: Alsughayer *et al.* (2011 ▶); Joshi & Khosla (2003 ▶); Scozzafava *et al.* (2003 ▶); Drews (2000 ▶); Peixoto & Beverley (1987 ▶). For crystal structures of closely related compounds, see: Boechat *et al.* (2010 ▶); Zia-ur-Rehman *et al.* (2009 ▶).
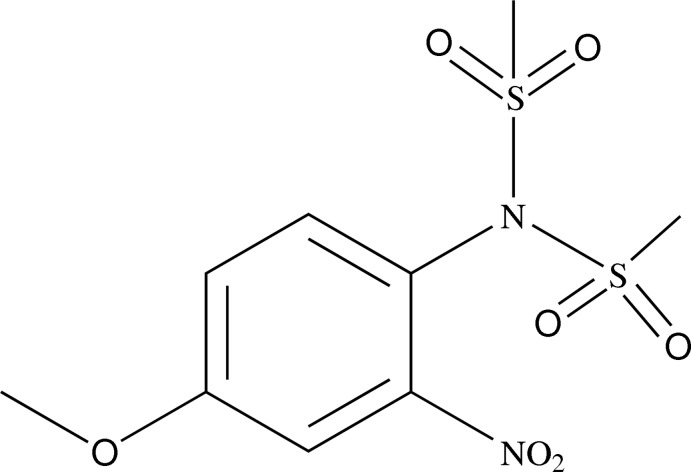


## Experimental

### 

#### Crystal data

C_9_H_12_N_2_O_7_S_2_
*M* *_r_* = 324.33
Monoclinic, 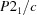

*a* = 9.4976 (7) Å
*b* = 7.5987 (6) Å
*c* = 19.2434 (15) Å
β = 103.672 (2)°
*V* = 1349.43 (18) Å^3^
*Z* = 4
Mo *K*α radiation
μ = 0.43 mm^−1^
*T* = 273 K
0.55 × 0.47 × 0.11 mm

#### Data collection

Bruker SMART APEX CCD area-detector diffractometer
Absorption correction: multi-scan (*SADABS*; Bruker, 2000 ▶) *T*_min_ = 0.799, *T*_max_ = 0.955
9504 measured reflections
3362 independent reflections
2711 reflections with *I* > 2σ(*I*)
*R*_int_ = 0.023

#### Refinement

*R*[*F* ^2^ > 2σ(*F* ^2^)] = 0.040
*wR*(*F* ^2^) = 0.113
*S* = 1.05
3362 reflections
185 parameters
H-atom parameters constrained
Δρ_max_ = 0.31 e Å^−3^
Δρ_min_ = −0.30 e Å^−3^

### 

Data collection: *SMART* (Bruker, 2000 ▶); cell refinement: *SAINT* (Bruker, 2000 ▶); data reduction: *SAINT*; program(s) used to solve structure: *SHELXS97* (Sheldrick, 2008 ▶); program(s) used to refine structure: *SHELXL97* (Sheldrick, 2008 ▶); molecular graphics: *SHELXTL* (Sheldrick, 2008 ▶); software used to prepare material for publication: *SHELXTL*, *PARST* (Nardelli, 1995 ▶) and *PLATON* (Spek, 2009 ▶).

## Supplementary Material

Crystal structure: contains datablock(s) global, I. DOI: 10.1107/S160053681202483X/is5148sup1.cif

Structure factors: contains datablock(s) I. DOI: 10.1107/S160053681202483X/is5148Isup2.hkl

Supplementary material file. DOI: 10.1107/S160053681202483X/is5148Isup3.cml

Additional supplementary materials:  crystallographic information; 3D view; checkCIF report

## Figures and Tables

**Table 1:** Hydrogen-bond geometry (Å, °)

*D*—H⋯*A*	*D*—H	H⋯*A*	*D*⋯*A*	*D*—H⋯*A*
C1—H1*B*⋯O2^i^	0.93	2.46	3.368 (2)	165
C8—H8*B*⋯O4	0.96	2.58	3.225 (3)	125
C8—H8*C*⋯O5^ii^	0.96	2.58	3.250 (3)	127
C9—H9*B*⋯O1	0.96	2.59	3.226 (3)	124
C9—H9*B*⋯O1^iii^	0.96	2.47	3.173 (3)	130

Symmetry codes: (i) 
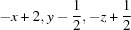
; (ii) 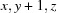; (iii) 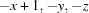.

**Fig. 1.**
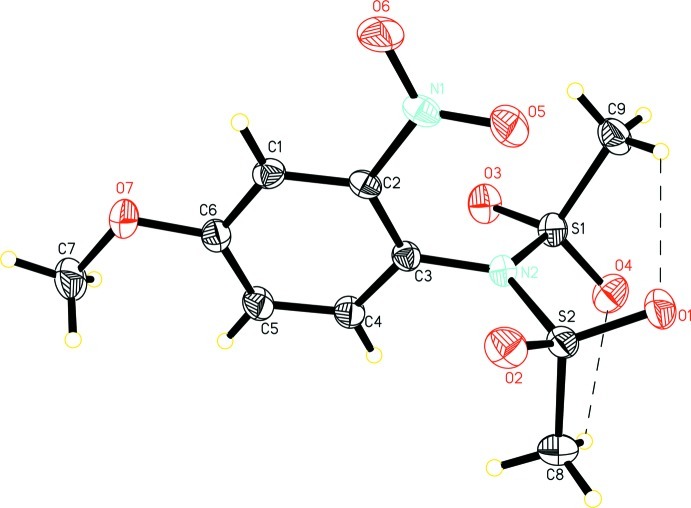
The molecular structure of the title compound with displacement ellipsoids drawn at 30% probability level.

**Fig. 2.**
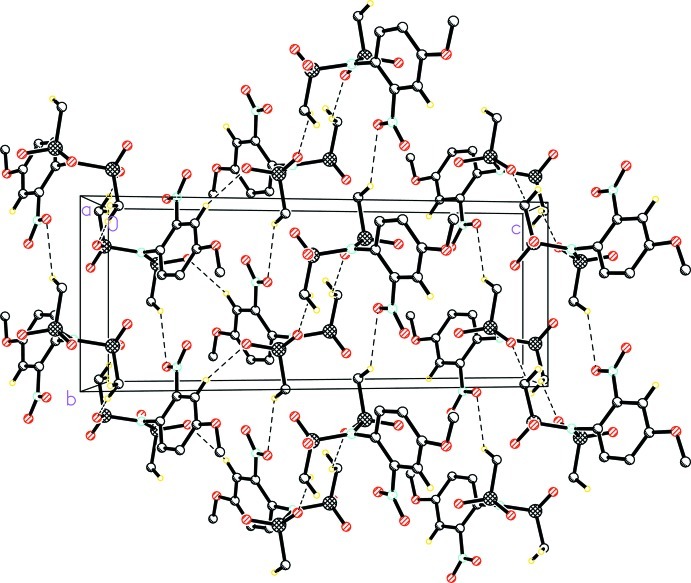
The crystal packing of the title compound. Only hydrogen atoms involved in hydrogen bonding are shown.

## supplementary crystallographic information

### Comment

Compounds containing the sulfonamide moiety have attracted a wide interest due to their interesting chemical and biological properties, which makes them promising candidates in drug discovery. Sulfonamides posses wide variety of biological activities including anti-bacterial, anti-leishmanial, anti-inflammatory, anti-cancer, and carbonic anhydrase inhibitory activities (Alsughayer *et al.*, 2011; Joshi & Khosla, 2003; Scozzafava *et al.*, 2003; Drews, 2000; Peixoto & Beverley, 1987). The title compound was prepared as a part of our ongoing research to synthesize different sulfonamide derivatives to study their bioactive potential and structure activity relationship (SAR). In the title compound (Fig. 1), the nitro group was found to be slightly twisted with the dihedral angle of 14.69 (10)° between the NO_2_ group and the benzene ring. The S1—N2—C3—C4 and S2—N2—C3—C4 torsion angles are 82.83 (19) and -92.42 (17)°, respectively. The molecule is stabilized by two intramolecular C8—H8B···O4 and C9—H9B···O1 interactions to form two *S*(6) ring motifs. In the crystal structure, the molecules are linked to form a two-dimensional network through C1—H1B···O2^i^, C8—H8C···O5^ii^ and C9—H9B···O1^iii^ intermolecular hydrogen bonds (Fig. 2 and Table 1). The bond lengths and angles are within the normal range and similar to other closley related structures (Boechat *et al.*, 2010; Zia-ur-Rehman *et al.*, 2009).

### Experimental

To a stirring solution of methanesulfonyl chloride (1.0 g, 8.7 mmol) in CH_2_Cl_2_ (20 ml) at 0 °C, 3 ml Et_3_N and 4-methoxy-2-nitroaniline (1.1 eq., 1.61 g m, 9.6 mmol) were added along with catalytic amount of dimethylamino pyridine (DMAP). Progress of the reaction was monitored by thin layer chromatography in 7:3 hexanes: ethyl acetate solvent system. After complete consumption of starting material (2 hrs), workup was performed with H_2_O (10 ml), organic layer was separated and aqueous layer was extracted with CH_2_Cl_2_ (2 × 10 ml). Organic layers were further washed with brine (10 ml), and dried over MgSO_4_, filtered, and concentrated in vacuum to obtain the crude product (0.9 g, 90% yield). Flash chromatography was performed hexanes: ethyl acetate (7:3), to obtain crystalline compound I, in 55% yield. Crystals were found suitable for single-crystal X-ray diffraction studies. All the starting materials and solvents were purchased from commercial suppliers and used for reaction without purification.

### Refinement

H atoms were positioned geometrically with C—H = 0.96 or 0.93 Å, and constrained to ride on their parent atoms with *U*_iso_(H)= 1.2*U*_eq_(C) or 1.5*U*_eq_(C_methyl_). A rotating group model was applied to the methyl groups.

### Crystal data

**Table:**

C_9_H_12_N_2_O_7_S_2_	*F*(000) = 672
*M**_r_* = 324.33	*D*_x_ = 1.596 Mg m^−^^3^
Monoclinic, *P*2_1_/*c*	Mo *K*α radiation, λ = 0.71073 Å
Hall symbol: -P 2ybc	Cell parameters from 3096 reflections
*a* = 9.4976 (7) Å	θ = 2.2–27.7°
*b* = 7.5987 (6) Å	µ = 0.43 mm^−^^1^
*c* = 19.2434 (15) Å	*T* = 273 K
β = 103.672 (2)°	Block, yellow
*V* = 1349.43 (18) Å^3^	0.55 × 0.47 × 0.11 mm
*Z* = 4	

### Data collection

**Table:**

Bruker SMART APEX CCD area-detector diffractometer	3362 independent reflections
Radiation source: fine-focus sealed tube	2711 reflections with *I* > 2σ(*I*)
Graphite monochromator	*R*_int_ = 0.023
ω scan	θ_max_ = 28.3°, θ_min_ = 2.2°
Absorption correction: multi-scan (*SADABS*; Bruker, 2000)	*h* = −12→12
*T*_min_ = 0.799, *T*_max_ = 0.955	*k* = −10→9
9504 measured reflections	*l* = −25→23

### Refinement

**Table:**

Refinement on *F*^2^	Secondary atom site location: difference Fourier map
Least-squares matrix: full	Hydrogen site location: inferred from neighbouring sites
*R*[*F*^2^ > 2σ(*F*^2^)] = 0.040	H-atom parameters constrained
*wR*(*F*^2^) = 0.113	*w* = 1/[σ^2^(*F*_o_^2^) + (0.0641*P*)^2^ + 0.2074*P*] where *P* = (*F*_o_^2^ + 2*F*_c_^2^)/3
*S* = 1.05	(Δ/σ)_max_ = 0.001
3362 reflections	Δρ_max_ = 0.31 e Å^−^^3^
185 parameters	Δρ_min_ = −0.30 e Å^−^^3^
0 restraints	Extinction correction: *SHELXL97* (Sheldrick, 2008), Fc^*^=kFc[1+0.001xFc^2^λ^3^/sin(2θ)]^-1/4^
Primary atom site location: structure-invariant direct methods	Extinction coefficient: 0.0101 (14)

### Special details

**Table:**

Geometry. All e.s.d.'s (except the e.s.d. in the dihedral angle between two l.s. planes) are estimated using the full covariance matrix. The cell e.s.d.'s are taken into account individually in the estimation of e.s.d.'s in distances, angles and torsion angles; correlations between e.s.d.'s in cell parameters are only used when they are defined by crystal symmetry. An approximate (isotropic) treatment of cell e.s.d.'s is used for estimating e.s.d.'s involving l.s. planes.
Refinement. Refinement of *F*^2^ against ALL reflections. The weighted *R*-factor *wR* and goodness of fit *S* are based on *F*^2^, conventional *R*-factors *R* are based on *F*, with *F* set to zero for negative *F*^2^. The threshold expression of *F*^2^ > σ(*F*^2^) is used only for calculating *R*-factors(gt) *etc*. and is not relevant to the choice of reflections for refinement. *R*-factors based on *F*^2^ are statistically about twice as large as those based on *F*, and *R*- factors based on ALL data will be even larger.

### Fractional atomic coordinates and isotropic or equivalent isotropic displacement parameters (Å^2^)

**Table:**

	*x*	*y*	*z*	*U*_iso_*/*U*_eq_	
S1	0.80706 (5)	0.21065 (6)	−0.02024 (2)	0.03866 (15)	
S2	0.67671 (5)	0.31016 (6)	0.09782 (2)	0.03825 (15)	
O1	0.55361 (15)	0.22596 (19)	0.05410 (8)	0.0529 (4)	
O2	0.71498 (16)	0.2788 (2)	0.17294 (7)	0.0514 (4)	
O3	0.95185 (15)	0.2109 (2)	−0.02879 (7)	0.0522 (4)	
O4	0.70649 (16)	0.3355 (2)	−0.05856 (7)	0.0561 (4)	
O5	0.80101 (17)	−0.07491 (19)	0.12618 (9)	0.0608 (4)	
O6	0.99411 (19)	−0.20742 (17)	0.18046 (9)	0.0602 (4)	
O7	1.36416 (14)	0.2232 (2)	0.25913 (8)	0.0503 (4)	
N1	0.92993 (18)	−0.07629 (18)	0.15472 (8)	0.0381 (3)	
N2	0.82155 (15)	0.24662 (18)	0.06736 (7)	0.0329 (3)	
C1	1.14724 (19)	0.0885 (2)	0.20463 (9)	0.0345 (4)	
H1B	1.1802	−0.0117	0.2313	0.041*	
C2	1.01332 (18)	0.0890 (2)	0.15816 (8)	0.0311 (3)	
C3	0.96028 (17)	0.2371 (2)	0.11724 (8)	0.0315 (3)	
C4	1.04925 (19)	0.3835 (2)	0.12521 (10)	0.0406 (4)	
H4B	1.0171	0.4833	0.0982	0.049*	
C5	1.1838 (2)	0.3863 (2)	0.17185 (10)	0.0428 (4)	
H5A	1.2407	0.4872	0.1764	0.051*	
C6	1.23354 (19)	0.2382 (3)	0.21179 (9)	0.0366 (4)	
C7	1.4561 (2)	0.3748 (3)	0.27101 (13)	0.0630 (6)	
H7A	1.5458	0.3458	0.3040	0.095*	
H7B	1.4090	0.4680	0.2905	0.095*	
H7C	1.4752	0.4123	0.2265	0.095*	
C8	0.6647 (3)	0.5369 (3)	0.08270 (13)	0.0576 (6)	
H8A	0.5830	0.5830	0.0981	0.086*	
H8B	0.6528	0.5598	0.0326	0.086*	
H8C	0.7517	0.5927	0.1091	0.086*	
C9	0.7335 (2)	−0.0003 (3)	−0.03754 (11)	0.0507 (5)	
H9A	0.7196	−0.0262	−0.0875	0.076*	
H9B	0.6421	−0.0054	−0.0246	0.076*	
H9C	0.7986	−0.0849	−0.0100	0.076*	

### Atomic displacement parameters (Å^2^)

**Table:**

	*U*^11^	*U*^22^	*U*^33^	*U*^12^	*U*^13^	*U*^23^
S1	0.0379 (3)	0.0453 (3)	0.0321 (2)	−0.00244 (18)	0.00691 (18)	0.00241 (17)
S2	0.0339 (2)	0.0363 (2)	0.0449 (3)	−0.00356 (16)	0.00994 (18)	−0.00732 (18)
O1	0.0338 (7)	0.0560 (9)	0.0673 (10)	−0.0104 (6)	0.0088 (6)	−0.0157 (7)
O2	0.0573 (9)	0.0568 (9)	0.0437 (8)	−0.0021 (7)	0.0191 (6)	−0.0065 (6)
O3	0.0448 (8)	0.0697 (10)	0.0460 (8)	−0.0059 (7)	0.0185 (6)	−0.0001 (7)
O4	0.0582 (9)	0.0632 (9)	0.0421 (7)	0.0097 (7)	0.0020 (6)	0.0133 (7)
O5	0.0512 (9)	0.0431 (8)	0.0834 (11)	−0.0183 (7)	0.0065 (8)	0.0071 (7)
O6	0.0801 (12)	0.0310 (7)	0.0648 (10)	−0.0038 (7)	0.0078 (8)	0.0130 (7)
O7	0.0351 (7)	0.0564 (9)	0.0529 (8)	−0.0008 (6)	−0.0029 (6)	−0.0013 (7)
N1	0.0530 (9)	0.0287 (7)	0.0343 (7)	−0.0084 (6)	0.0137 (6)	−0.0001 (6)
N2	0.0306 (7)	0.0356 (7)	0.0310 (7)	−0.0017 (6)	0.0044 (5)	−0.0005 (6)
C1	0.0407 (9)	0.0316 (8)	0.0311 (8)	0.0033 (7)	0.0082 (6)	0.0031 (6)
C2	0.0388 (9)	0.0254 (8)	0.0307 (8)	−0.0054 (6)	0.0114 (6)	−0.0010 (6)
C3	0.0323 (8)	0.0300 (8)	0.0317 (8)	−0.0036 (6)	0.0062 (6)	0.0016 (6)
C4	0.0416 (10)	0.0297 (8)	0.0466 (10)	−0.0064 (7)	0.0029 (7)	0.0078 (7)
C5	0.0403 (10)	0.0347 (9)	0.0508 (11)	−0.0112 (7)	0.0054 (8)	0.0039 (8)
C6	0.0336 (9)	0.0417 (9)	0.0337 (9)	−0.0008 (7)	0.0065 (7)	−0.0032 (7)
C7	0.0358 (11)	0.0725 (15)	0.0743 (15)	−0.0108 (10)	0.0002 (10)	−0.0152 (13)
C8	0.0612 (14)	0.0352 (10)	0.0733 (15)	0.0068 (9)	0.0096 (11)	−0.0051 (10)
C9	0.0521 (12)	0.0529 (12)	0.0456 (11)	−0.0100 (9)	0.0089 (9)	−0.0160 (9)

### Geometric parameters (Å, º)

**Table:**

S1—O4	1.4223 (15)	C1—H1B	0.9300
S1—O3	1.4231 (14)	C2—C3	1.397 (2)
S1—N2	1.6809 (14)	C3—C4	1.383 (2)
S1—C9	1.748 (2)	C4—C5	1.377 (2)
S2—O1	1.4210 (14)	C4—H4B	0.9300
S2—O2	1.4247 (14)	C5—C6	1.382 (3)
S2—N2	1.6882 (15)	C5—H5A	0.9300
S2—C8	1.747 (2)	C7—H7A	0.9600
O5—N1	1.218 (2)	C7—H7B	0.9600
O6—N1	1.211 (2)	C7—H7C	0.9600
O7—C6	1.359 (2)	C8—H8A	0.9600
O7—C7	1.431 (3)	C8—H8B	0.9600
N1—C2	1.478 (2)	C8—H8C	0.9600
N2—C3	1.437 (2)	C9—H9A	0.9600
C1—C2	1.371 (2)	C9—H9B	0.9600
C1—C6	1.389 (2)	C9—H9C	0.9600
			
O4—S1—O3	119.21 (9)	C5—C4—C3	122.23 (16)
O4—S1—N2	107.30 (8)	C5—C4—H4B	118.9
O3—S1—N2	105.27 (8)	C3—C4—H4B	118.9
O4—S1—C9	108.87 (10)	C4—C5—C6	119.50 (17)
O3—S1—C9	109.43 (10)	C4—C5—H5A	120.3
N2—S1—C9	105.93 (9)	C6—C5—H5A	120.3
O1—S2—O2	120.15 (9)	O7—C6—C5	125.38 (17)
O1—S2—N2	106.83 (8)	O7—C6—C1	114.93 (17)
O2—S2—N2	105.73 (8)	C5—C6—C1	119.70 (16)
O1—S2—C8	109.48 (11)	O7—C7—H7A	109.5
O2—S2—C8	108.96 (10)	O7—C7—H7B	109.5
N2—S2—C8	104.52 (10)	H7A—C7—H7B	109.5
C6—O7—C7	117.75 (16)	O7—C7—H7C	109.5
O6—N1—O5	123.14 (15)	H7A—C7—H7C	109.5
O6—N1—C2	117.92 (15)	H7B—C7—H7C	109.5
O5—N1—C2	118.94 (14)	S2—C8—H8A	109.5
C3—N2—S1	120.43 (11)	S2—C8—H8B	109.5
C3—N2—S2	118.43 (11)	H8A—C8—H8B	109.5
S1—N2—S2	120.96 (8)	S2—C8—H8C	109.5
C2—C1—C6	119.80 (16)	H8A—C8—H8C	109.5
C2—C1—H1B	120.1	H8B—C8—H8C	109.5
C6—C1—H1B	120.1	S1—C9—H9A	109.5
C1—C2—C3	121.66 (15)	S1—C9—H9B	109.5
C1—C2—N1	115.57 (14)	H9A—C9—H9B	109.5
C3—C2—N1	122.77 (15)	S1—C9—H9C	109.5
C4—C3—C2	117.11 (15)	H9A—C9—H9C	109.5
C4—C3—N2	118.25 (15)	H9B—C9—H9C	109.5
C2—C3—N2	124.63 (15)		
			
O4—S1—N2—C3	−139.57 (14)	C1—C2—C3—C4	0.4 (2)
O3—S1—N2—C3	−11.63 (15)	N1—C2—C3—C4	−179.79 (15)
C9—S1—N2—C3	104.25 (14)	C1—C2—C3—N2	179.70 (15)
O4—S1—N2—S2	35.55 (12)	N1—C2—C3—N2	−0.5 (3)
O3—S1—N2—S2	163.50 (10)	S1—N2—C3—C4	82.83 (19)
C9—S1—N2—S2	−80.63 (12)	S2—N2—C3—C4	−92.42 (17)
O1—S2—N2—C3	−150.32 (13)	S1—N2—C3—C2	−96.42 (17)
O2—S2—N2—C3	−21.24 (15)	S2—N2—C3—C2	88.33 (19)
C8—S2—N2—C3	93.69 (15)	C2—C3—C4—C5	−0.8 (3)
O1—S2—N2—S1	34.46 (12)	N2—C3—C4—C5	179.87 (17)
O2—S2—N2—S1	163.53 (10)	C3—C4—C5—C6	0.7 (3)
C8—S2—N2—S1	−81.53 (12)	C7—O7—C6—C5	2.6 (3)
C6—C1—C2—C3	0.0 (3)	C7—O7—C6—C1	−177.73 (17)
C6—C1—C2—N1	−179.76 (15)	C4—C5—C6—O7	179.46 (18)
O6—N1—C2—C1	−14.8 (2)	C4—C5—C6—C1	−0.2 (3)
O5—N1—C2—C1	165.47 (17)	C2—C1—C6—O7	−179.86 (15)
O6—N1—C2—C3	165.42 (17)	C2—C1—C6—C5	−0.2 (3)
O5—N1—C2—C3	−14.3 (2)		

### Hydrogen-bond geometry (Å, º)

**Table:**

*D*—H···*A*	*D*—H	H···*A*	*D*···*A*	*D*—H···*A*
C1—H1*B*···O2^i^	0.93	2.46	3.368 (2)	165
C8—H8*B*···O4	0.96	2.58	3.225 (3)	125
C8—H8*C*···O5^ii^	0.96	2.58	3.250 (3)	127
C9—H9*B*···O1	0.96	2.59	3.226 (3)	124
C9—H9*B*···O1^iii^	0.96	2.47	3.173 (3)	130

Symmetry codes: (i) −*x*+2, *y*−1/2, −*z*+1/2; (ii) *x*, *y*+1, *z*; (iii) −*x*+1, −*y*, −*z*.
